# High clinical heterogeneity in a Chinese pedigree of retinal vasculopathy with cerebral leukoencephalopathy and systemic manifestations (RVCL-S)

**DOI:** 10.1186/s13023-021-01712-9

**Published:** 2021-01-30

**Authors:** Nina Xie, Qiying Sun, Jinxia Yang, Yangjie Zhou, Hongwei Xu, Lin Zhou, Yafang Zhou

**Affiliations:** 1 Department of Geriatric Neurology, Xiangya Hospital, Central South University, Changsha, 410008 China; 2 National Clinical Research Center for Geriatric Disorders, Changsha, 410078 Hunan China

**Keywords:** Hereditary cerebral small vessel disease, Clinical neurology, Neurogenetics, Neuro-ophthalmology, Neuro-radiology

## Abstract

**Background:**

Being a newly defined disease, RVCL-S is underrecognized by clinicians globally. It is an autosomal dominantly inherited small vessel disease caused by the heterozygous C-terminal frameshift mutation in *TREX1* gene. RVCL-S is featured by cerebral dysfunction, retinopathy, and vasculopathy in multiple internal organs. Misdiagnosis may cause devastating consequences in patients, such as iatrogenic PML caused by misuse of immunosuppressants. Thus, increasing awareness of this disease is in urgent need.

**Results:**

We uncovered a large Chinese origin RVCL-S pedigree bearing the *TREX1* mutation. A comprehensive characterization combining clinical, genetic, and neuropathological analysis was performed. The Intrafamilial comparison showed highly heterogeneous clinical phenotypes. Mutation carriers in our pedigree presented with retinopathy (8/13), seizures (2/13), increased intracranial pressure (1/13), mild cognitive impairment (3/13), stroke-like episode (3/13), mesenteric ischemia (1/13), nephropathy (9/13), ascites (3/13), hypertension (9/13), hyperlipidemia (3/8), hypoalbuminemia (3/8), normocytic anemia (3/8), subclinical hypothyroidism (1/8), hyperfibrinogenemia (1/8), hyperparathyroidism (2/8), and abnormal inflammatory markers (4/8). The constellation of symptoms is highly varied, making RVCL-S a challenging diagnosis. Comparison with reported RVCL-S pedigrees further revealed that the mesenteric ischemia is a novel clinical finding and the MRS pattern of brain lesions is emulating neoplasm and tumefactive demyelination.

**Conclusion:**

Our reports characterize a highly heterogeneous RVCL-S pedigree, highlight the probability of misdiagnosis in clinical practice, and broaden the clinical spectrum of RVCL-S.

## Introduction

Retinal vasculopathy with cerebral leukoencephalopathy and systemic manifestations (RVCL-S) is an autosomal dominantly inherited small vessel disease caused by the heterozygous C-terminal frameshift mutation in *TREX1* gene [1]. It is used to be named as cerebroretinal vasculopathy (CRV), hereditary vascular retinopathy (HVR), hereditary systemic angiopathy (HSA), and hereditary endotheliopathy, retinopathy, nephropathy and stroke (HERNS) according to the organ of dominant involvement. Since the gene was identified in 2007, the previous seemingly unrelated clinical manifestations in diverse organs whose functioning depends on intact microvascularity, such as eye, brain, and kidney, are reconciled here as a systemic disorder. *TREX1* has only one exon. The encoded protein TREX1 is a DNA-specific 3′–5′ exonuclease, widely expressed in mammalian cells. Small as it is, *TREX1* plays a pivotal role in DNA repair, protein glycosylation, and innate immune regulation. The exonuclease function of mutant TREX1 is not affected. Mutant TREX1 may cause subcellular mislocalization, glycosylation defects, and dysregulated innate immune regulation, leading to the wide array of symptoms in RVCL-S. To our knowledge, twenty-one genetically confirmed pedigrees and cases had been reported worldwide. The majority of them are of Caucasian ethnicity. Of note, Netherland alone possesses three RVCL-S pedigrees, a country with only 17 million inhabitants, suggesting an underestimation of its prevalence [1–12, 22, 23, 26–30].

Clinical features commonly associated with RVCL-S have included cerebral dysfunction, retinopathy, nephropathy, hepatic abnormalities, hypertension, anemia, gastrointestinal symptoms, subclinical hypothyroidism, and Raynaud phenomenon [1–3]. Histopathology is characterized by vasculopathy of multiple organs and ultra-structurally multi-laminated vascular basement membranes [4–6]. These non-specific features overlap with a myriad of ischemic disorders. Confirmative diagnosis of RVCL-S relies on genetic testing. Intriguingly, RVCL-S has high phenotypic variability. The development of retinopathy, cerebral dysfunction, and other systemic manifestations is asynchronous. The constellation between them has no fixed mode. The severity of organ damage differs from patient to patient. For example, renal disease development and Raynaud’s phenomenon can precede core symptoms affecting the eyes and brain. Brain MRI features can vary from non-specific white matter hyperintensity to tumefactive lesions. A mutation carrier may be asymptomatic or die prematurely. Exact mechanisms underlying this phenomenon need further elucidation. Due to its rarity and heterogeneity, RVCL-S is usually misdiagnosed as brain tumors, tumefactive demyelination, and organ disease of unknown origin, leading to misusage of drugs and unnecessary invasive procedures. Thus, increasing awareness of this disease is in urgent need. Recently, we uncovered a large pedigree of Chinese origin. A comprehensive strategy combining clinical, genetic, and neuropathological analysis was adopted.

## Methods

### Patients and family members

**Fig. 1:**
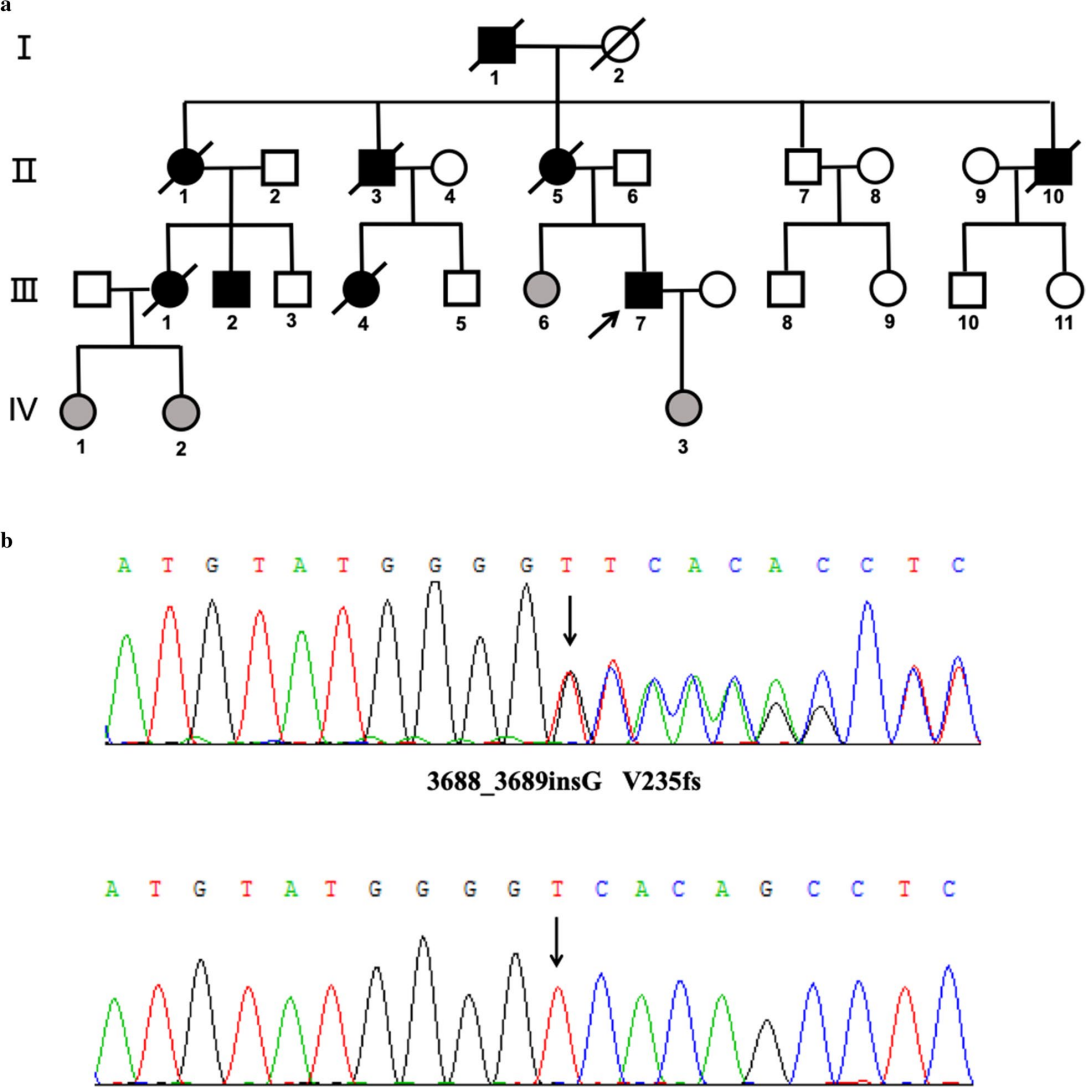
Pedigree and sequencing result. Black colored shapes indicate affected individuals. Gray-colored shapes indicate pre-manifest mutation carriers. Shapes with oblique lines represent the departed

We investigated a large family with 26 members spanning four generations, originating from Hunan province, China (Fig. 1a). Thirteen family members including the proband (III-7) carried a heterozygous mutation in the *TREX1* gene. Family members without a *TREX1* mutation were included as controls.

### Clinical, pathological, and genetic evaluation

Elements of this study involving human subjects were approved by the Medical Ethics Committee of Xiangya Hospital, Central South University. All participants provided informed written consent before initiating research and data collection. History-taking, physical examination, blood tests(regular blood tests, liver function, renal function, serum lipid, coagulation test, ESR, CRP, IL-6, thyroid, and parathyroid function), urine and stool analysis, radiology(unenhanced CT, gadolinium enhanced T1 weighted image, T2 weighted image, FLAIR, DWI, and MRS), and ophthalmic examinations(fluorescein fundus angiography and optical coherence tomography) were performed either prospectively or retrospectively for mutation carriers III-1,2,4,6,7. Other mutation carriers were interviewed remotely, and clinical data were obtained when possible. For patient 3(III-4), tumor resection surgery was performed for the right frontal lesion. Post-operatively, brain tissues were formalin fixed, sectioned, stained with hematoxylin & eosin, and immunostained for microscopic observation. DNA was extracted from peripheral blood using the QIAamp DNA investigator kit (QIAGEN, USA). Sanger sequencing was done as previously reported for all family members [1].

## Results

### Clinical findings

#### Patient 1 (III-7, proband) has multisystemic manifestations involving eye, brain, kidney, and mesentery

**Fig. 2:**
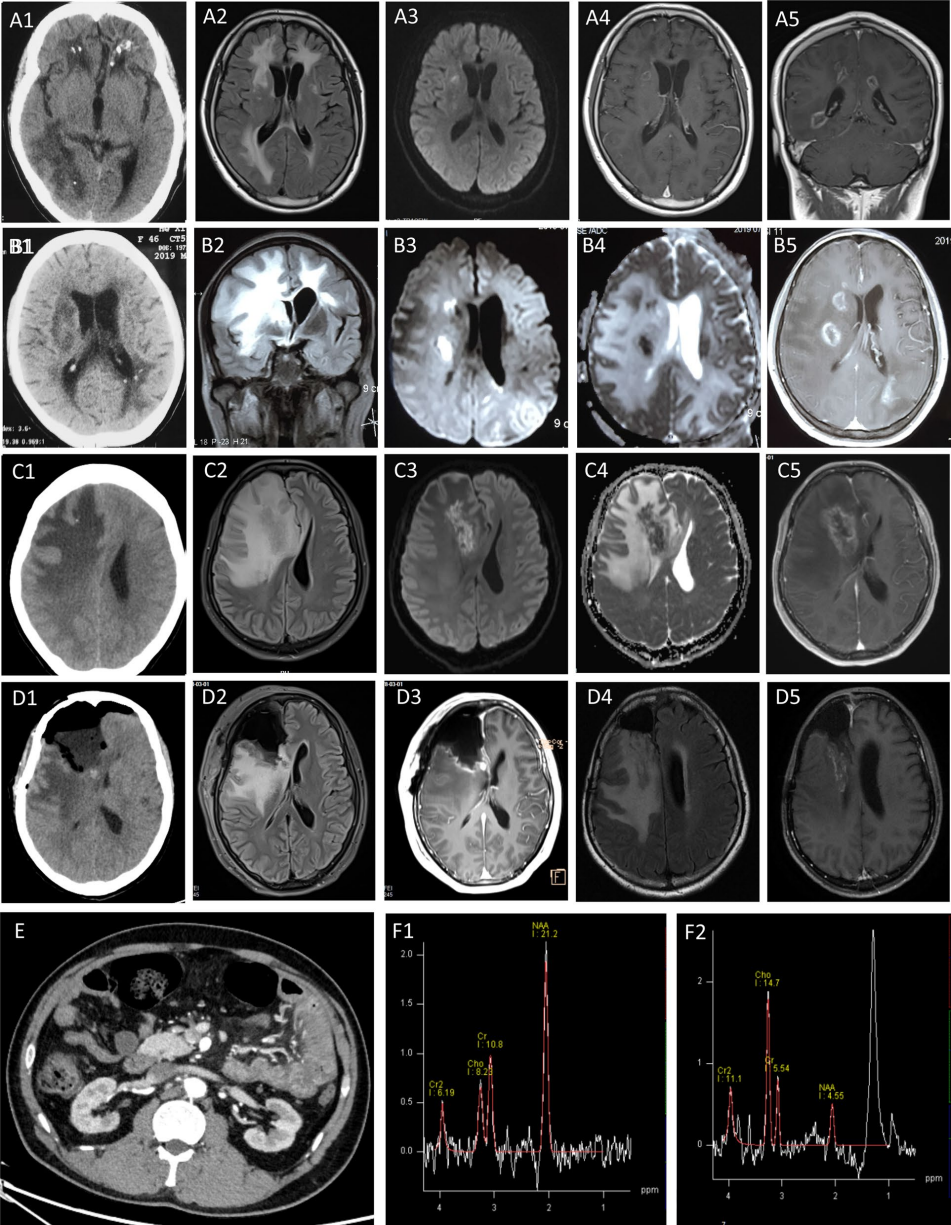
Radiological tests. **a** brain CT and MRI of the proband (III-7). (**A1**) Non-contrast CT scan showed extensively hypodense lesions with focal calcifications. (**A2**) FLAIR images showed diffuse leukoencephalopathy bilaterally. (**A3**) DWI indicated central restricted diffusion. (**A4**, **A5**) Post-contrast MRI revealed multiple irregular rim-enhancing lesions. **b** brain CT and MRI of patient 2 (III-1). (**B1**) Non-contrast CT scan showed extensively hypodense lesions with focal calcifications. (**B2**) Coronary FLAIR images showed diffuse leukoencephalopathy. (**B3**, **B4**) DWI paired with ADC indicated central restricted diffusion. (**B5**) Post-contrast MRI showed multiple irregular rim-enhancing lesions in the periventricular region. **c** Preoperative neuroimages of patient 3 (III-4). (**C1**) Non-contrast CT scan showed irregular hypodense lesions in the right frontal lobe and basal ganglia region with mass effect and a tiny calcified dot. (**C2**) FLAIR images showed iso- to hyperintense lesions with peripheral edema. (**C3**, **C4**) DWI paired with ADC indicated central restricted diffusion. (**C5**) Post-contrast MRI showed a garland-like enhancement pattern. **d** Postoperative neuroimages of patient 3 (III-4). (**D1**) Non-contrast CT scan showed postoperative changes in the residual cavity. (**D2**, **D3**) FLAIR and enhanced MRI done immediately after the surgery showed removal of the lesion and enhancement of the surrounding meninges. (**D4**, **D5**) One month later, a follow-up MRI showed newly occurred rim-enhancing lesions posterior to the resected region, with residual cavity shrank and meningeal enhancement diminished, which was misdiagnosed as radiation-induced necrosis by the radiologist. **e** Abdominal CT of the proband (III-7). Post-contrast CT showed segmental intestinal wall edema in the mid-upper abdomen and mesenteric inflammatory changes (edema, panniculitis, increased blood vessel density, and vessel dilatation). **f** MRS pattern. (**F1**) normal tissue. (**F2**) a ring-enhancing lesion of patient 3 (III-4)

**Fig. 3:**
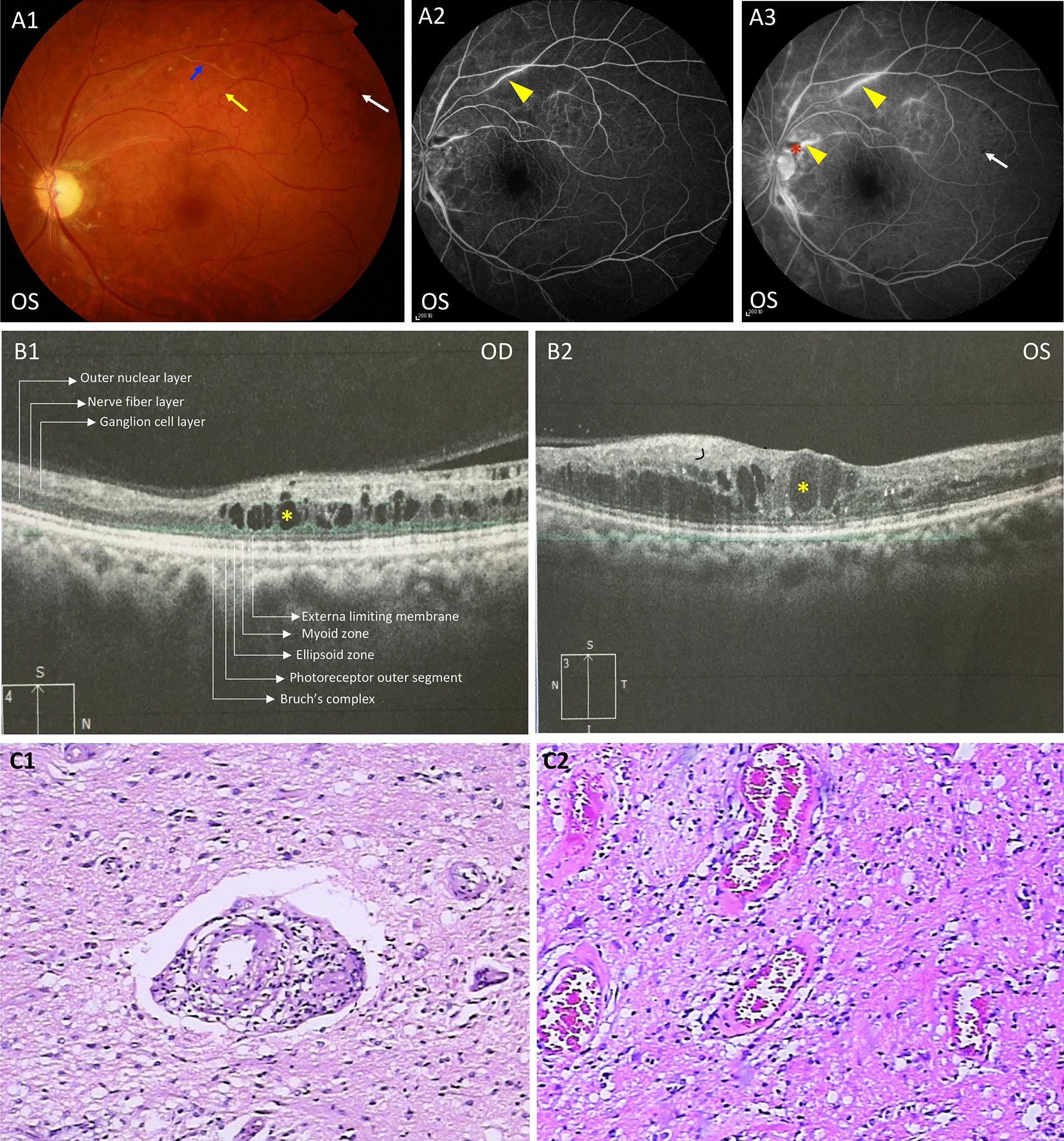
Ophthalmic findings and neuropathology. **a** Funduscope and FFA of the proband (III-7). Vascular sheathing was shown by the blue arrow. Vein tortuosity was shown by the yellow arrow. Perivascular fluorescein leakage and hyper-fluorescence of vessel walls were shown by yellow arrowheads. Non-perfusion area was shown by the red asterisk. Microbleeds were shown by white arrows. **b** OCT results of patient 2 (III-1). Cystoid intraretinal fluid pockets were shown by the yellow asterisk. **c** Neuropathology and immunostaining of patient 3 (III-4). Neuropathology demonstrated focal necrosis, gliosis, lumen stenosis, and lymphocytic infiltration within the vessel wall (100×). Immunostaining was positive for GFAP, Olig2, CD3, CD20, CD68, and CD138, and negative for Ki67, P53, IDH1, PAX-5, IgG, and IgG4. Specific stain by Congo red was negative

A 35-year-old man presented to us because of being attacked by a generalized tonic–clonic seizure three days ago. He had a history of worsening eyesight for six years as well as chronic kidney disease (CKD) and hypertension for three years. Physical examination was unremarkable except for blurred vision (OD 20/50, OS 20/40) and mild cognitive impairment(MMSE = 24, MOCA = 21). Lab workup results were significant for proteinuria (3.47 g/24 h, ref < 0.15 g/24 h), hyperlipidemia, renal insufficiency (creatinine 288 umol/L, ref 41–111 umo/L; BUN 11.11 mmol/L, ref 3.1–8.0 mmol/L), hyperfibrinogenemia (Fibrinogen 5.79 g/L, ref 2.0–4.0 g/L), and elevated inflammatory markers (ESR 86 mm/h, ref 0–15 mm/h; CRP 9.3 mg/L, ref 0–8 mg/L; 5.98 g/L, ref 2.0–4.0 g/L; FDP 34.9 mg/L, ref 0–5 mg/L; D-dimer 7.71 mg/L, ref 0–0.5 mg/L). Estimated GFR was 38.5 mL/min (ref GFR > 80 mL/min). Non-contrast head CT showed extensive subcortical hypodense lesions with punctate calcifications. Enhanced brain MRI revealed diffuse leukoencephalopathy and multiple rim-enhancing lesions (Fig. 2a). Cerebral spinal fluid(CSF) profile was normal. Ophthalmoscope observed optic atrophy. Fluorescein fundus angiography (FFA) reported retinal vasculopathy (Fig. 3a). Given the family history and simultaneous involvement of eye, brain, and kidney, the diagnosis of RVCL-S was suspected, which was further confirmed by genetic testing. Eight months later, he developed left limb weakness and recurrent abdominal pain. Repeated brain MRI showed expansion of white matter hyperintensities. Enhanced abdominal CT indicated chronic mesenteric ischemia (Fig. 2e and Additional file 1: Fig. S1). Cilostazol was given to improve microvascular circulation.

#### Patient 2 (III-1) has similar multisystemic manifestations to the proband

A 46-year-old woman presented to us because of subacute-onset slurred speech, dysphagia, and left limb weakness for two months. She was diagnosed with renal insufficiency four years ago. Upon neurological examination, she had decreased vision (OD 20/63, OS 20/100). Left arm strength was rated 2/5, left leg strength was rated 5-/5, both distally and proximally, with brisk tendon reflexes and negative Babinski’s sign. Lab test results disclosed normocytic anemia (105 g/L, ref 115–150 g/L), subclinical hypothyroidism (TSH 7.749 uIU/mL, ref 0.35–4.94 uIU/mL), increased ESR (55 mm/h, ref 0–15 mm/h), decreased C3 (574.0 mg/L, ref 790–1520 mg/L), increased D-dimer (0.69 mg/L, ref 0–0.5 mg/L) and FDP (7.0 mg/L, ref 0–5 mg/L), proteinuria (urine protein ++), hypoalbuminemia (36.5 g/L, ref 40.0–55.0 g/L), hyperlipidemia (LDL 3.75 mmol/L, ref 1.55–3.19 mmol/L), increased creatinine (316.4 umol/L, ref 41–111.0 umo/L), and increased BUN (12.87 mmol/L, ref 2.6–7.5 mmol/L). Estimated GFR was 15.03 mL/min. Neuroimaging findings were featured by diffuse leukoencephalopathy with multiple rim-enhancing lesions and punctate calcifications (Fig. 2b). OCT showed cystoid intraretinal fluid pockets in both eyes (Fig. 3b). Diagnosis of RVCL-S was further confirmed by genetic testing.

#### Patient 3 (III-4) was misdiagnosed as glioma and deceased post-surgery

This sister came to our hospital when she was 39 years old because of a 20-day history of intermittent headache accompanied by projectile vomiting and left arm convulsion. Past medical history was unremarkable. Neurological examination was notable for blurred vision and bilateral abduction deficits. Preoperative lab tests revealed hypoalbuminemia (28.6 g/L, ref 40.0–55.0 g/L), increased BUN (8.99 mmol/L, ref 2.60–7.50 mmol/L), normocytic anemia (92.0 g/L, ref 115.0–150.0 g/L), as well as elevated FDP (5.8 g/L, ref 2.0–4.0 g/L) and D-dimer (0.25 mg/L, ref 0–0.5 mg/L). Estimated GFR was 41.49 mL/min. Non-contrast head CT showed irregular hypodense lesions in the right frontal lobe and basal ganglia region. Brain MRI with gadolinium revealed a 54 × 40 × 24 mm tumefactive lesion which had a garland-like enhancement pattern (Fig. 2c). Magnetic resonance spectroscopy (MRS) showed decreased N-acetyl aspartate levels, increased Choline levels, and lipid peaks (Fig. 2f). She was suspected of glioblastoma. Tumor resection surgery was implemented. Postoperative brain MRI with gadolinium showed removal of the lesion (Fig. 2d). However, neuropathology showed vasculopathy with mild-to-moderate inflammatory changes. There is focal necrosis, gliosis, lumen stenosis, and lymphocytic infiltration within the vessel wall (100×). Immunostaining was positive for GFAP, Olig2, CD3, CD20, CD68, and CD138, and negative for Ki67, P53, IDH1, PAX-5, IgG, and IgG4. Specific stain by Congo red was negative (Fig. 3c). One month after discharge, she was hospitalized again because of sudden-onset hemiplegia on the left side. Physical examination revealed 2/5 strength of the left extremities. Enhanced brain MRI showed newly occurred rim-enhancing lesions posterior to the resected region (Fig. 2d). Prednisolone was administered. She was discharged two weeks later with the left extremities recovered to 4/5 strength. Unfortunately, she passed away at home for unknown causes one year later.

#### Patient 4 (III-2) indicates that nephropathy can be the sole presentation of RVCL-S

This brother was referred to our hospital when he was 41 years old because of a one-year history of fatigue. Past medical history was notable for a 3-month history of hypertension. Physical examination revealed conjunctival pallor and positive shifting dullness. Lab test results disclosed normocytic anemia (110 g/L, ref 130–175 g/L), proteinuria (1.47 g/24 h), hypoalbuminemia (33.1 g/L, ref 40.0–55.0 g/L), hyperlipidemia, increased creatinine (511 umol/L, ref 53–132.6 umo/L), increased BUN (10.63 mmol/L, ref 2.9–7.14 mmol/L), decreased 25-hydroxyvitamin D (14.35 ng/mL, ref > 20 ng/mL), increased parathyroid hormone (248.7 pg/mL, ref 15–65 pg/mL), as well as elevated ESR and IL-6 level (47 mm/h, ref 0–15 mm/h; 7.6 ng/mL, ref 0–7 ng/mL). Estimated GFR was 9.6 mL/min. He was diagnosed with chronic renal failure, renal hypertension, anemia, ascites, and secondary hyperparathyroidism. Peritoneal dialysis and supportive treatment were administered. Genetic testing confirmed that he was also a mutation carrier of RVCL-S.

#### Genetic analysis showed patients in the pedigree were caused by a *TREX1* mutation

13 of 26 members are mutation carriers of the C-terminal frameshift mutation V235fs (3688_3689insG), a known mutation as previously reported (Fig. 1b) [1].

#### A RVCL-S pedigree with highly variable intrafamilial phenotypes

Clinical features were summarized in Table 1. The mean age of onset was 37.2 (range 29–42). The mean age at diagnosis was 40.4 (range 35–46). All deceased members died in their 40 s. The mean age of death was 43.1 (range 40–47). Symptoms and severity vary from person to person. I-1, II-1,3,5,10 died of unexplained kidney disease. III-4 (patient 3) is misdiagnosed as glioma. III-7 (the proband) and III-1 (patient 2) have multisystemic involvement. III-2 (patient 4) solely has kidney involvement. III-6, IV-1, IV-2, and IV-3 are pre-manifest mutation carriers.

**Table 1:** Clinical feature summary of the Chinese origin RVCL-S pedigree

	I-1	II-1	II-5	II-3	II-10	III-1	III-2	III-4		III-6		III-7	IV-1	IV-2	IV-3
*Demographics*															
Age at onset						42	40	39				29			
Age at diagnosis						46	41	39		41		35	26	19	5
Age at death	42	45	43	41	44	47		40							
Gender	M	F	F	M	M	F	M	F		F		M	F	F	F
*Symptoms and signs*															
Retinopathy	+	+	+	+	+	+	−	+		−		+	−	−	−
Seizures	−	−	−	−	−	−	−	+		−		+	−	−	−
ICP						−	−	+		−		−	−	−	−
Cognitive decline	+					+	−	−		−		+	−	−	−
Stroke-like episode	−	−	−	−	−	+	−	+		−		+	−	−	−
Nephropathy	+	+	+	+	+	+	+	+		−		+	−	−	−
Mesenteric ischemia						−	−	−		−		+	−	−	−
Ascites	+					−	+	−		−		+	−	−	−
Hypertension	+	+	+	+	+	+	+	+		−		+	−	−	−
*Lab workups*															
Proteinuria						+	+	+		−		+	−	−	−
GFR (mL/min)						15.03	9.6	41.49		Normal		38.5	Normal	Normal	Normal
Hyperlipidemia						+	+	−		−		+	−	−	−
Hypoalbuminemia						+	+	+		−		−	−	−	−
Normocytic anemia						+	+	+		−		−	−	−	−
Hyperfibrinogenemia						−	−	−		−		+	−	−	−
Hypothyroidism						+	−	−		−		−	−	−	−
Hyperparathyroidism						−	+	−		−		−	−	−	−
Inflammatory markers						ESR, FDP, D-D↑	IL-6, ESR↑	FDP, D-D↑		Normal		ESR, CRP, FDP, D-D↑	Normal	Normal	Normal
*Neuroimagings*															
Leukoaraosis						Fazekas 3		Fazekas 1		Fazekas 1		Fazekas 3	None	None	None
Number of rim-enhancing lesion						Multiple		Single		None		Multiple			
Mass effect and edema						+		+		None		+	None	None	None
*Neuropathology*								vasculopathy mimicking CNS vasculitis							

^a^Empty cells lack relevant information

Upon history-taking and physical examination, mutation carriers had retinopathy (8/13), seizures (2/13), increased intracranial pressure (1/13), mild cognitive impairment (3/13), stroke-like episode (3/13), mesenteric ischemia (1/13), nephropathy (9/13), ascites (3/13), and hypertension (9/13). Lab workups revealed renal insufficiency (4/8), hyperlipidemia (3/8), hypoalbuminemia (3/8), normocytic anemia (3/8), subclinical hypothyroidism (1/8), hyperfibrinogenemia (1/8), hyperparathyroidism (2/8), and abnormal inflammatory markers (4/8). IL-6, ESR, CRP, FDP, and D-dimer were significantly elevated in the more severely affected mutation carriers (III-1,2,4,7) compared to the pre-manifest mutation carriers (III-6, IV-1, IV-2, IV-3) and controls.

Neuroimaging tests were done in seven mutation carriers (III-1, 4, 6, 7 and IV-1, 2, 3). Unenhanced brain CT and enhanced MRI uncovered the features of diffuse white matter hyperintensities (2/7), non-specific white matter hyperintensities (1/7), calcifications (3/7), multiple rim-enhancing lesions (2/7), and a single large tumefactive lesion (1/7), the neuropathology of which indicated vasculopathy. The MRS pattern of the rim-enhancing lesions showed decreased NAA peak, increased Cho peak, and lipid peak presence. These features highly emulate tumefactive demyelination (TD) and glioma (Fig. 2). To aid differential diagnosis in clinical settings, we compared the neuroimaging features between RVCL-S, TD, and glioma in Table 2.

**Table 2:** comparison of neuroimaging features between RVCL-S, TD, and glioma

	CT	T1WI	T2WI/FLAIR	Diffusion restriction	Gd +	MRS	Other adjuncts
RVCL-S	Hypodense	Hypointense	Hyperintense	Central	Irregular rim	NAA ↓	Family history ( +)
	± calcification		Mixed		Punctate	Cho↑	Multisystemic symptoms ( +)
					Nodular	Lip peak	PET/CT may show decreased glucose uptake
					None		CSF profiling is normal
							Decreased cerebral blood perfusion on PWI
TD	Hypodense	Hypointense	Peripheral hypointense rim with central hyperintense signal	Peripheral	Open ring (more common)	NAA ↓	Ring enhancement co-localizing with T2W rim
		Mixed			Closed ring	Cho↑	Multiple ovoid lesions perpendicular to lateral ventricles
					Homogeneous		
					Patchy		Optic neuritis is more common than retinopathy
					Cotton-ball		OCB is more likely to be positive
					Nodular		PET/CT may show increased glucose uptake at the rim of the lesion with central hypometabolism
					None		Decreased cerebral blood perfusion on PWI
Glioma	Hypodense	Hypointense	Hyperintense	Heterogeneous central	Closed ring (more common)	NAA ↓	High cerebral blood perfusion on PWI
	Hyperdense	Isointense	Isointense	Incomplete peripheral	Central homogeneous	Cho↑	Hemorrhagic lesion may present
	Mixed	Mixed	Mixed		Central heterogeneous	Lip peak	PET/CT usually show increased glucose uptake
	± calcification						

#### Comparison between RVCL-S pedigrees reveals novel clinical findings

Major features of genetically confirmed RVCL-S pedigrees and cases are summarized in Table 3. To our knowledge, nine frameshift mutations have been found so far. The majority of them are reported in Caucasians. Only two pedigrees are of Chinese origin. The same mutation can yield distinctive clinical phenotypes in different pedigrees and mutation carriers in the same pedigree can have different clinical manifestations. Generally, clinical manifestations of our pedigree are consistent with previously reported. The majority of patients have involvement in eye, brain, and kidney. In addition, our pedigree revealed a relatively younger age at death, a novel clinical phenotype of mesenteric ischemia, and a first description of MRS pattern for the brain lesions.

**Table 3:** Comparison between RVCL-S pedigrees and individual patients

Origin	American	European	Chinese	Jewish	Dutch	German	Australian	Italian	Caucasian	Mexican	Japanese	Chinese
		American	American	American				Australian				
Families or patients	4 patients	1 family	1 family	1 patient	3 families	1 family	1 family	1 family	1 family	1 family	1 family	1 family
	1family								2 patients		2 patients	
Mutation(s)	D278Efs*48	V235fs	T249fs	V235fs	V235fs	T236fs	R284fs	V235fs	V235fs	V235fs	T249fs	V235fs
	V235fs				L287fs				T270fs		V235fs	
	E285*								P275Qfs*2			
	T249fs											
*Clinical manifestations*												
Focal neurological deficits	+	+	+	+	+	+	+	+	+	+	+	+
Cognitive decline	+	+	+	+	+	+	+	+	+			+
Seizures		+		+	+		+		+			+
Migraine		+	+	+	+		+		+	+		
Psychiatric symptoms		+	+	+	+	+	+		+			
Retinopathy	+	+	+	+	+	+	+	+	+	+	+	+
Liver abnormality	+	+		+	+	+	+	+	+			
Nephropathy	+	+	+	+	+	+	+		+	+	+	+
Gastrointestinal bleeding		+		+	+	+			+			
Mesenteric ischemia												+
Anemia	+	+		+	+		+					+
Hypertension		+	+	+	+		+		+	+		+
Raynaud’s phenomenon		+		+	+							
Subclinical hypothyroidism					+							+
Cardiomyopathy	+								+			
Osteonecrosis									+			
Skin lesions					+							
*Neuroimaging features*												
T2 White matter lesions	+	+	+	+	+	+	+	+	+	+	+	+
Cerebral calcification	+	+	+	+	+		+		+		+	+
Rim-like enhancement	+	+	+	+	+	+	+	+	+	+		+
Nodular enhancement		+	+	+	+	+	+	+				
Abnormal MRS pattern												+
*Histopathology*												
Cerebral vasculopathy with mild-to-moderate inflammatory infiltrate	+	+	+		+	+	+	+	+	+	+	+
Peripheral neuropathy									+			
Myopathy									+			
Arteriolo-nephrosclerosis	+		+						+		+	
Hepatic fibrosis or nodular regenerative hyperplasia						+			+			
Leucocytoclastic vasculitis and multilaminated capillary basement membranes			+					+	+			
References	[2, 4, 7, 9, 10]	[4, 25]	[4, 5]	[4]	[1, 3, 26]	[4, 22]	[4, 27]	[4, 12]	[6, 8, 11]	[23]	[28–30]	Our report

## Discussion

Here we describe a Chinese origin RVCL-S pedigree carrying the V235fs mutation in *TREX1*. The intrafamilial comparison revealed highly heterogeneous clinical phenotypes, including unaffected pre-manifest mutation carrier, single organ damage, multisystemic involvement, and premature death. This phenomenon implicates that RVCL-S should not be immediately excluded even in the absence of family history and early diagnosis is challenging when the patient only shows subtle symptoms in a single organ.

Another observation from intrafamilial comparison is the abnormal level of inflammatory markers. TREX1 is not only an exonuclease but an anti-inflammatory protein. Single-stranded DNA(ssDNA) stimulates type1 interferon-associated inflammatory response to clean out invaded pathogens. TREX1 degrades ssDNA to prevent pathological immune activation [7, 8]. Mutations abolishing TREX1 enzymatic activity will result in autoimmune activation and type1 interferon upregulation, causing Aicardi-Goutières syndrome, familial Chilblain Lupus, and systemic lupus erythematosus [9–11]. Although direct evidence showing accumulation of aberrant ssDNA in RVCL-S patients is lacking, autoantibodies against non-nuclear antigens are significantly elevated in the mice model conditionally expressing V235fs, suggesting autoimmune activation may also contribute to RVCL-S pathogenesis [12]. Our reports support this view by showing that inflammatory markers, including FDP, D-dimer, ESR, CRP, and IL-6, are significantly elevated in severely affected patients compared to the pre-manifest mutation carriers and controls.

Comparison with reported RVCL-S pedigrees revealed a relatively younger age at death, a novel clinical phenotype of mesenteric ischemia, and a cerebral MRS pattern emulating neoplasm and TD. Firstly, in the largest retrospective study of RVCL-S, mutation carriers died at a mean age of 53.1 years (range 32–72) and on average 9.0 years (range < 1–26) after diagnosis [4]. In our pedigree, mutation carriers died at a mean age of 43.1 years (range 40–47), on average 1.0 year after diagnosis, implicating under-recognition of this disorder and diagnostic delay in real clinical settings. Additionally, the previously reported digestive disorder is confined to gastrointestinal bleeding. Therefore the chronic mesenteric ischemia in the proband is a novel clinical finding. Mesenteric ischemia is a rare emergency condition to be neglected. If not diagnosed and treated promptly, some patients with advanced mesenteric ischemia may develop into sepsis and irreversible bowel infarction. Albeit lack of definitive pathological evidence, it is important to keep in mind the diagnosis of mesenteric ischemia based on clinical manifestation and abdominal CT examination. Lastly, the MRS pattern of RVCL-S brain lesions overlaps with that of tumor and TD. Each of them can show decreased NAA peak, increased Cho peak, and lipid peak presence. Over-reliance on MRS pattern may lead to a misdiagnosis.

Moreover, comparison with reported RVCL-S pedigrees revealed two interesting phenomenons. The first one is that mutation carriers in the same pedigree can have different clinical manifestations and the same mutation can yield distinctive clinical phenotypes in different pedigrees. The mechanisms underlying this heterogeneity is a key unanswered question. We assume that epigenetics may contribute to RVCL heterogeneity. Studies on epigenetics are needed to shed light on this topic. The other one is that most patients have involvement in three organs including the eye, brain, and kidney. The reason why there is a predilection for eye, brain, and kidney in RVCL-S remains an enigma. Interestingly, this phenomenon is not exclusive to RVCL-S. Chronic kidney disease and retinopathy have been linked with cerebral small vessel disease caused by other etiologies, such as diabetes mellitus and hypertension. A prevailing view is that vasoregulation of the microvasculatures of these organs is similar both anatomically and functionally. For example, small vessels branching from large high-pressure arteries, such as the glomerular afferent arterioles and cerebral perforating arteries, have to maintain strong vascular tone to create a large gradient in a short distance, making them more susceptible to injury [13–15]. Below we would like to briefly discuss the differential diagnosis of retinopathy, nephropathy, and cerebral rim-enhancing lesions.

Retinal vasculopathy is easily misdiagnosed as diabetic/hypertensive retinopathy and retinal vasculitis [2, 4, 16], owing to their overlapping funduscopic features. For example, FFA results of patient 1 (III-7) and patient 2 (III-1) are consistently misinterpreted as retinal vasculitis throughout the disease course. The coexistence of inflammatory infiltration (Vascular sheathing), vessel wall damage (microaneurysms, perivascular fluorescein leakage, and microbleeds), and ischemic changes (non-perfusion area, dilated tortuous veins, and telangiectasia) is misleading even to an experienced ophthalmologist.

Patient 4 (III-2) is an example of RVCL-S exclusively presenting as CKD of unknown cause. Although this patient refused renal biopsy, this case and others highlight the likelihood of unnecessary renal biopsy in RVCL-S patients [6, 17, 18]. Recently another RVCL-S case with renal impairment being the sole presentation was reported, emphasizing the importance of differentiating RVCL-S from thrombotic microangiopathy (TMA), a systemic disorder with a predilection for kidney. The renal pathology of RVCL-S is characterized by arterio- and arteriolar nephrosclerosis, duplicated glomerular basement membranes, and microthrombi in glomerular capillaries, mimicking both chronic and acute components of TMA. Several red flags are worthy of being noted. Firstly, extrarenal manifestations of TMA, including hemolysis and thrombocytopenia, are uncommon for RVCL-S. Additionally, therapeutics for TMA, such as anti-complement therapy and plasma change, are not effective to RVCL-S [18].

In RVCL-S, the white matter hyperintensities on brain MRI could be focal, confluent, or tumefactive, occasionally associated with calcifications. The enhancement pattern is variable, including rim-like, nodular, or none. The most predominant MRI feature shared by our patients is the rim-enhancing lesion, which is highly likely to be confused with radiation-induced necrosis, infection, tumefactive demyelination (TD), and glioma. It is relatively more difficult to distinguish RVCL-S from TD and glioma in clinical practice, especially in the absence of family history. For each of them can show lesions of T1W hypointensity, T2W hyperintensity, and rim-like enhancement under certain circumstances. Their MRS patterns are also highly similar. Patient 2 (III-1) and previous reports demonstrated that misdiagnosing RVCL-S as TD or glioma will lead to unnecessary brain surgery and misusage of immunomodulators. The subsequent destruction of functional brain tissues and iatrogenic progressive multifocal leukoencephalopathy (PML) may cause devastating effects [2, 16, 19–21].

As direct radiological comparison between RVCL-S, TD, and glioma is lacking, according to available publications, we summarized several distinguishable neuroimaging features in Table 2 [22–28]. Firstly, we assume that unenhanced CT, DWI, and PWI are ponderable adjuncts. On CT scan, hypodensity with calcification is a feature of RVCL-S but not TD. Hemorrhage is common for glioma but unusual for RVCL-S. On DWI, RVCL-S lesions tend to have central diffusion restriction. TD is more likely to have peripheral diffusion restriction. On PWI, RVCL-S and TD are hypo-perfused due to vasculopathy and the paucity of neo-vessels, while glioma is hyper-perfused. Besides, the constellation of certain features is helpful. For example, a T2W homogenous center with a complete hypointense rim which co-localizes with an enhanced ring is an observation supporting TD. Last but not least, other parameters, including a detailed review of systemic manifestations, radiotherapy history, family history, physical examination, CSF analysis, oligoclonal bands, specimen culture, and 18F-FDG PET/CT, should not be neglected.

We emphasize that timely and accurate diagnosis of RVCL-S is to avoid unnecessary invasive procedures. However, diagnostic procedures sometimes may inevitably come to this step. Brain biopsy or surgery was recommended in selected patient as following: the patient presents with obvious increased intracranial pressure or cerebral hernia; there is refractory symptom such as long-term epilepsy, which could not be controlled by drug; the lesion is located in an easily accessible area for operation with tremendously differential diagnosis difficulty between RVCL-S and glioma. Neuropathologists should reconsider the diagnosis when there is no unequivocal evidence for demyelination or neoplasm. *TREX1* sequencing should be considered when facing patients with cerebral rim-enhancing lesions and multisystemic involvement. Multidisciplinary team (MDT) consultation, including neurologist, neurosurgeon, urologist, ophthalmologist, pathologist, genetic counselor, and radiologist, will be helpful when there is a diagnostic dilemma.

Unfortunately, no effective curative treatment is available. Glucocorticoids may alleviate vasogenic cerebral edema for tumor-like lesions temporarily [2, 5, 21]. The use of immunosuppressants(cyclophosphamide, methotrexate, azathioprine), plasma change, and IVIg may not be favorable [6, 29, 30]. With the rationale of reducing endothelium damage and microthrombi formation. Anti-coagulation and anti-platelet therapy have been trialed in several cases and shown to stabilize the disease transiently. However, the therapeutic effects are inconsistent among studies [6, 17]. We tried the PDE III inhibitor, cilostazol, in patient 1 (III-7) when he developed mesenteric ischemia. Subsequent symptomatic alleviation suggested cilostazol might be a potential therapeutic strategy. Whether type 1 interferon-α contributes to RVCL-S pathogenesis remains controversial, so does the feasibility of anti-interferon therapy [16, 18, 20]. Other immunotherapies, such as natalizumab, tofacitinib, and hydroxychloroquine, are hypothesized to have beneficial effects. However, successful practical experience is lacking due to complications [2, 21]. As aforementioned, in our pedigree, the inflammatory markers' level is significantly higher in the symptomatic mutation carriers compared to the asymptomatic ones. Our findings suggest the pathogenic role played by neuroinflammation and support the view that anti-inflammatory therapy is worth trying. Aclacinomycin, an OST complex inhibitor, was shown to have therapeutic potential in animal models [31]. This observation has spawned a phase I clinical trial for aclarubicin. Evaluating the long-term effects of these therapeutic strategies is of great significance.

## Conclusion

In sum, our reports characterize a highly heterogeneous RVCL-S pedigree, highlight the probability of misdiagnosis in clinical practice, and broaden the clinical spectrum of RVCL-S.

## Supplementary Information

**Additional file 1. Fig. 1**: Enhanced abdominal CT of patient 1(III-7). (A) Coronary and (B-E) axial sections showing segmental intestinal wall edema in the mid-upper abdomen and mesenteric inflammatory changes (edema, panniculitis, increased blood vessel density, blurred vessel walls, and vessel dilatation). The yellow arrowhead indicates mild stenosis in the upper mesenteric artery.

## Data Availability

The datasets used and/or analysed during the current study are available from the corresponding author on reasonable request.

## Abbreviations

BUN: Blood urea nitrogen
CSF: Cerebral spinal fluid
CRP: C-reactive protein
CKD: Chronic kidney disease
Cho: Choline
ESR: Erythrocyte sedimentation rate
FDP: Fibrinogen degradation product
FFA: Fluorescein fundus angiography
FLAIR: Fluid attenuated inversion recovery
18F-FDG PET/CT: 18F-fluorodeoxyglucose positron emission tomography/computerized tomography
GFR: Glomerular filtration rate
MRS: Magnetic resonance spectroscopy
NAA: N-acetylaspartate
OCT: Optical coherence tomography
OCB: Oligoclonal bands
PML: Progressive multifocal leukoencephalopathy
PWI: Perfusion weighted imaging
RVCL-S: Retinal vasculopathy with cerebral leukoencephalopathy and systemic manifestations
ssDNA: Single-stranded DNA
TD: Tumefactive demyelination
TMA: Thrombotic microangiopathy
T1WI: T1 weighted image
T2WI: T2 weighted image
