# Supplementary figures and images for: High clinical heterogeneity in a Chinese pedigree of retinal vasculopathy with cerebral leukoencephalopathy and systemic manifestations (RVCL-S)
Source: Orphanet J Rare Dis. 2021 Jan 30;16:56. doi: 10.1186/s13023-021-01712-9 (PMC7847589; doi:10.1186/s13023-021-01712-9)

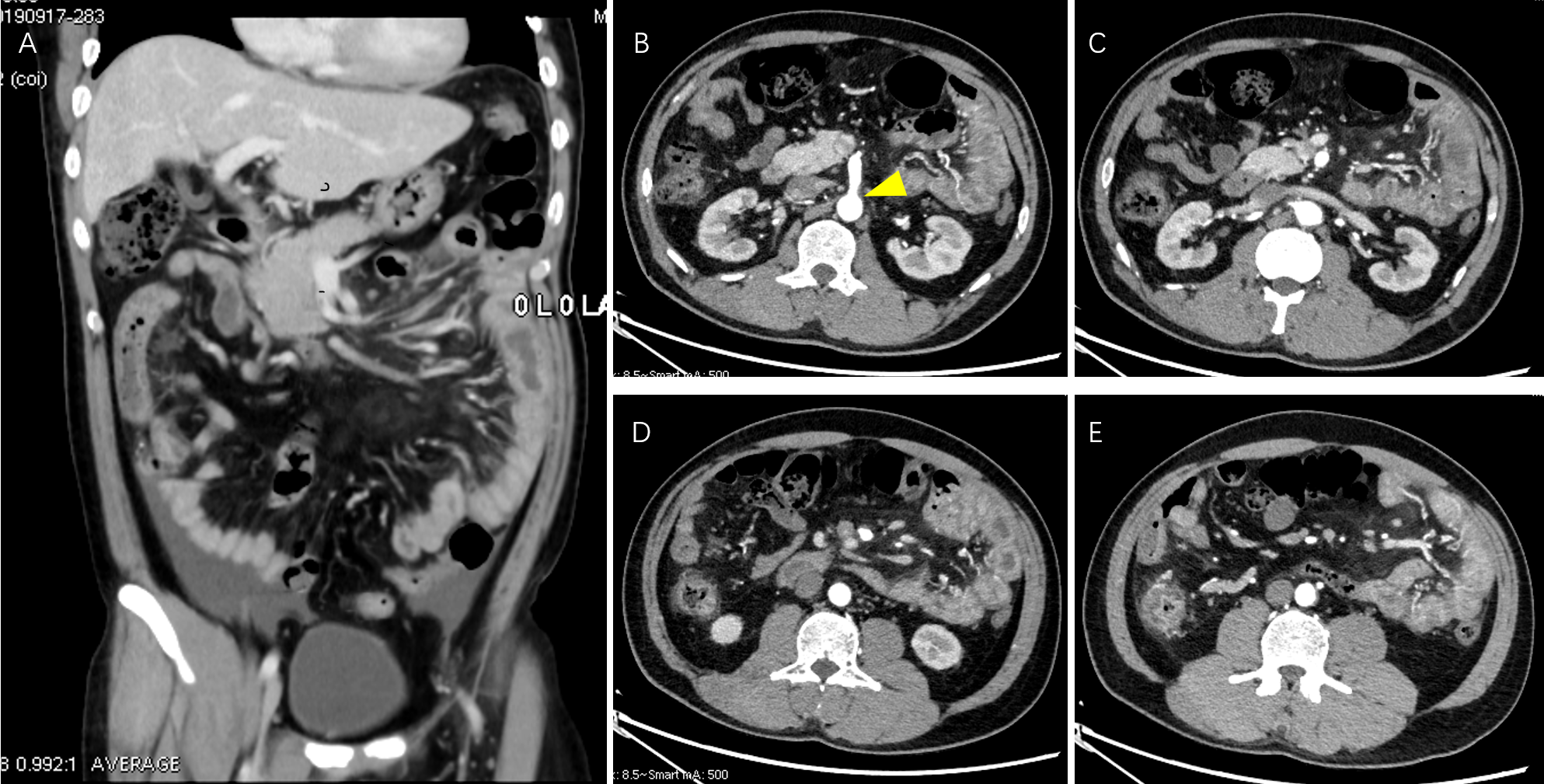

Supplement: Supplementary file 1 — Additional file 1. Fig. 1: Enhanced abdominal CT of patient 1(III-7). (A) Coronary and (B-E) axial sections showing segmental intestinal wall edema in the mid-upper abdomen and mesenteric inflammatory changes (edema, panniculitis, increased blood vessel density, blurred vessel walls, and vessel dilatation). The yellow arrowhead indicates mild stenosis in the upper mesenteric artery. [file 13023_2021_1712_MOESM1_ESM.png]
